# Point-driven modern Chladni figures with symmetry breaking

**DOI:** 10.1038/s41598-018-29244-6

**Published:** 2018-07-18

**Authors:** P. H. Tuan, Y. H. Lai, C. P. Wen, K. F. Huang, Y. F. Chen

**Affiliations:** Department of Electrophysics, National Chiao Tung University, 1001 Ta-Hsueh Rd., Hsinchu, 30010 Taiwan

## Abstract

Point-driven modern Chladni figures subject to the symmetry breaking are systematically unveiled by developing a theoretical model and making experimental confirmation in the orthotropic brass. The plates with square shape are employed in the exploration based on the property that the orientation-dependent elastic anisotropy can be controlled by cutting the sides with a rotation angle with respect to the characteristic axes of the brass. Experimental results reveal that the orientation symmetry breaking not only causes the redistribution of resonant frequencies but also induces more resonant modes. More intriguingly, the driving position in some of new resonant modes can turn into the nodal point, whereas this position is always the anti-node in the isotropic case. The theoretical model is analytically developed by including a dimensionless parameter to consider the orientation symmetry-breaking effect in a generalized way. It is numerically verified that all experimental resonant frequencies and Chladni patterns can be well reconstructed with the developed model. The good agreement between theoretical calculations and experimental observations confirms the feasibility of using the developed model to analyze the modern Chladni experiment with orientation symmetry breaking. The developed model is believed to offer a powerful tool to build important database of plate resonant modes for the applications of controlling collective motions of micro objects.

## Introduction

Chladni sound figures of vibrating plates which greatly impressed Napoleon in 18^th^ century^1^ have inspired many essential research in modern physics such as quantum chaos^2^, self-organization of granular media^3,4^, microscale acoustofluidics^5,6^, and pattern formation^7,8^. Due to its advantages of robustness, low cost, easier observation, and high replicability, the historic vibrating plate experiment still serves as a promising candidate to develop frontier applications including automated patterning of micro-objects^9,10^, non-contaminated positioning of biomolecules^11,12^, and sorting different particles^13,14^. Nowadays the traditional violin-bowing method has been replaced by an oscillating point driving source in modern Chladni systems to provide more stable and reliable experimental results^15^. The point-driven modern Chladni figures have not only been broadly used to demonstrate wave physics in popular science but also applied to the groundbreaking technology of manipulating multi-object motion by a single-actuator system^13^.

**Figure 1:**
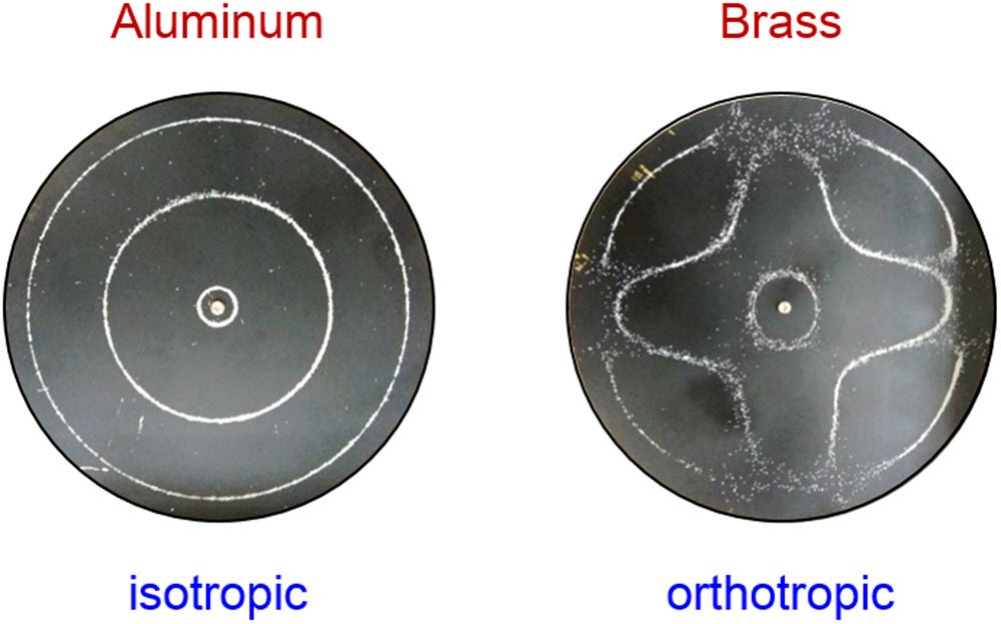
Comparison of typical Chladni figures for circular plates. The resonant Chladni figure of the isotropic aluminum plate follows the symmetry of the boundary geometry, whereas the case for the orthotropic brass cannot be simply explained by its boundary shape.

Recently, the formation of modern Chladni figures has been explicitly resolved by an analytical model considering the coupling effect between the thin plate and the point oscillator^16^. However, the current model only focuses on analyzing isotropic plates and cannot fully characterize the vibration of general systems with anisotropic properties. Unlike the isotropic cases, Chladni figures of anisotropic plates have been found to show morphologies with broken-symmetry that cannot be simply explained from the plate geometry as seen the comparison for aluminum and brass circular systems (Fig. 1). Even though the symmetry-breaking features in vibrating plates have been observed and explored for a long time^17–20^, a complete model that can nicely formulate all resonant spectra and Chladni figures for anisotropic systems remains highly desirable so far. Since the elastic anisotropy ubiquitously exists in materials such as copper, brass, silicon, sapphire, etc., which are commonly used in industry and semiconductor engineering, developing a general model to analyze modern Chladni figures for systems with broken orientation symmetry is greatly important to improve accuracy of actuating devices for micro-particles.

In this study, point-driven modern Chladni system subject to orientation symmetry breaking is thoroughly explored by developing a generalized model and making experimental confirmation in the orthotropic brass plates. Thanks to the orthotropy of brass^21^, plates with different elastic anisotropy magnitude can be directly made by cutting the brass sheet into squares with their sides along different rotation angles with respect to the characteristic axes of brass. Experimental results of the frequency spectra reveal that the increasing symmetry breaking not only arouses the redistribution of resonant peaks but also induces more resonant modes. More intriguingly, it is discovered that the driving position in some new resonant modes will turn into a nodal point, whereas this position is always an antinode for the isotropic plates. The peculiar morphology of new resonant modes with a nodal-point driving position is originated from antiphase superposition of nearly degenerate eigenstates which can only happen in systems with broken-symmetry^22^. By including a dimensionless parameter to consider the orientation symmetry-breaking effect in a generalized way, a theoretical model is analytically developed to reconstruct all experimental observations. The numerical reconstructions verify that all experimental resonant frequency spectra and Chladni figures can be satisfactorily described by the developed model. The good agreement between theoretical calculations and experimental results confirms the feasibility of using the developed model to efficiently analyze the vibrating modes and to effectively determine some critical elastic parameters of the anisotropic plates to greatly benefit various applications in practice.

## Results

### Modelling modern Chladni systems with symmetry breaking by orthotropic plates

The theoretical foundations for orthotropic systems are considered first to offer more general concepts for modern Chladni figures subject to orientation symmetry breaking. Note that in addition to orientation symmetry, plate systems possess translational symmetry which will also affect the resonant modes significantly if it is broken. However, studying the effect of translational symmetry breaking on plate resonance is beyond the scope of this work since the plates used in the experiments are considered to be uniform. The governed equation for the vibration mode *ψ* of orthotropic thin plates with two characteristic axes can be given by the anisotropic Kirchhoff-Love equation as^23^

1 $${D}_{x}\frac{{\partial }^{4}\psi }{\partial {x}^{4}}+2({\nu }_{xy}\,{D}_{y}+2G)\frac{{\partial }^{4}\psi }{\partial {x}^{2}\partial {y}^{2}}+{D}_{y}\frac{{\partial }^{4}\psi }{\partial {y}^{4}}-{\omega }^{2}\rho h\psi =0,$$

where *ν*_*xy*_ is the Poisson ratio in *xy*-plane, *G* is the in-plane shear modulus, ${D}_{x,y}={E}_{x,y}\,{h}^{3}/12(1-{\nu }_{xy}{\nu }_{yx})$ is the flexural rigidity, and ${E}_{x,y}$ is the Young’s modulus along the *x* or *y* characteristic direction. Note that the condition of symmetry of stiffnesses for orthotropic plates ensures ${\nu }_{xy}{E}_{y}={\nu }_{yx}{E}_{x}$. Due to the orthotropic property, bending waves inside the plate correspond to different acoustic speeds along different propagating directions. To determine the dispersion relation depending on the propagating orientation in the orthotropic plate, the plane-wave solution given by $\psi (x,y)={\psi }_{0}\,{e}^{-i{K}_{P}\cos \theta \cdot x}\,{e}^{-i{K}_{P}sin\theta \cdot y}$ with the amplitude of *ψ*_0_ and the propagating wave number ${K}_{P}(\theta )$ along an arbitrary direction with a rotation angle *θ* to one of the characteristic axes can be considered. Substituting the plane-wave solution into Eq. (1), the orientation-dependent dispersion relation of the orthotropic plate under the infinite plate approximation can be found to be

2 $${K}_{P}(\theta )={(\frac{{\omega }^{2}\rho h}{B(\theta )})}^{1/4}\,,$$

where

3 $$B(\theta )={D}_{x}{\cos }^{4}\theta +2({\nu }_{xy}{D}_{y}+2G){\cos }^{2}\theta {\sin }^{2}\theta +{D}_{y}{\sin }^{4}\theta .$$

Equations (2) and (3) clearly show that bending waves propagating along the directions denoted by *θ* and *θ* + π/2 to the orthotropic characteristic axes will more or less correspond to different acoustic speed except for the case with *θ* = π/4. Based on this property, the orientation symmetry breaking induced by the elastic anisotropy for the system can be flexibly adjusted by cutting orthotropic plates into squares with their sides along different angles *θ* with respect to the characteristic axes. More specifically, for an orthotropic square plate with the sides along *θ* and *θ* + π/2 directions, the quantitative measure for orientation symmetry breaking of the system can be simply related to the ratio between the propagating wave numbers as

4 $$\frac{{K}_{P}(\theta )}{{K}_{P}(\theta +\pi /2)}={[\frac{B(\theta +\pi /2)}{B(\theta )}]}^{1/4}=\frac{1-\delta (\theta )}{1+\delta (\theta )},$$

where $\delta (\theta )$ is a dimensionless symmetry-breaking parameter modelling the magnitude of elastic anisotropy and can be reversely evaluated as

5 $$\delta (\theta )=|\frac{1-{[B(\theta +\pi /2)/B(\theta )]}^{1/4}}{1+{[B(\theta +\pi /2)/B(\theta )]}^{1/4}}|.$$

Once *θ* is specified, the anisotropic Kirchhoff-Love equation given by Eq. (1) can be subsequently solved to find out the eigenmodes and eigenvalues for constructing the response wave function of the system. However, solving the vibration of free-edge plates has long been a tough problem even for the seemingly simple isotropic square systems^22^. Hence some critical assumptions are required to obtain an analytical expression for approximating the vibration wave function. Typically, the anisotropy of the orthotropic plate is dominated more by the different Young’s moduli for the two characteristic axes than by the Poisson effect. Besides, the in-plane shear effect is comparatively small for the vibrating thin plates. Therefore, the cross term for the coupling between *x* and *y* directions in Eq. (1) may be neglected. Consequently, the vibrating modes of orthotropic plates can be approximated by straightforwardly considering the overall anisotropic properties with the dimensionless symmetry-breaking parameter *δ* as

6 $${(1-\delta )}^{2}\frac{{\partial }^{4}\psi }{\partial {x}^{4}}+{(1+\delta )}^{2}\frac{{\partial }^{4}\psi }{\partial {y}^{4}}-{K}^{4}\,\psi =0,$$

where the effective wavenumber *K* includes contributions from *x*- and *y*-propagations as ${K}^{4}={({K}_{x}^{2}+{K}_{y}^{2})}^{2}$. Even though Eq. (6) still cannot be solved analytically for the square plate with free edges, its corresponding mode functions have been confirmed by Rayleigh^22^ that can be nicely approximated by the eigenfunctions of free-boundary membrane as long as the wavelength of bending wave is far larger than the thickness of plate^24^. Neglecting the cross term and assuming *x*- and *y*-coordinates of the system can be separable once again, the eigenmodes ${\psi }_{{n}_{1},{n}_{2}}(x,y)$ and eigenvalues ${K}_{{n}_{1},{n}_{2}}$ of the orthotropic square plate with the region in $0\le x,y\le a$ under the free-edge condition can be approximately given by

7 $${\psi }_{{n}_{1},{n}_{2}}(x,y)=\frac{2}{a}\,\cos (\frac{{n}_{1}\,\pi }{a}x)\cos (\frac{{n}_{2}\,\pi }{a}y)$$

and

8 $${K}_{{n}_{1},{n}_{2}}^{2}={(\frac{\pi }{a})}^{2}[(1-\delta ){{n}_{1}}^{2}+(1+\delta ){{n}_{2}}^{2}].$$

Note that ${n}_{1}=0,\,1,\,2,\ldots$ and ${n}_{2}=0,\,1,\,2,\ldots$ are respectively the mode indices along *x* and *y* coordinates of the plate. Using the approximated eigenmodes and eigenvalues, vibrating wave functions of point-driven square plate subject to orientation symmetry breaking can be generalized from previous work^16^ as

9 $${\rm{\Psi }}(x,y\,\,;\omega )=\sum _{{n}_{1},{n}_{2}}{C}_{{n}_{1},{n}_{2}}(x^{\prime} ,y^{\prime} ,\omega )\,\cos (\frac{{n}_{1}\,\pi }{a}x)\cos (\frac{{n}_{2}\,\pi }{a}y)$$

with

10 $${C}_{{n}_{1},{n}_{2}}(x^{\prime} ,y^{\prime} ,\omega )=\frac{4\,\,Q\,\,({m}_{d}/{m}_{p})}{1+\alpha \,\,\Xi (x^{\prime} ,y^{\prime} \,\,;\omega )}\,{(\frac{\omega }{{\omega }_{0}})}^{2}\frac{\cos (\frac{{n}_{1}\,\pi }{a}x^{\prime} )\cos (\frac{{n}_{2}\,\pi }{a}y^{\prime} )}{{[(1-\delta ){n}_{1}^{2}+(1+\delta ){n}_{2}^{2}]}^{2}-{[(\omega -i\gamma )/{\omega }_{o}]}^{2}}$$

and

11 $${\rm{\Xi }}(x^{\prime} ,y^{\prime} \,;\omega )=4{(\frac{\omega }{{\omega }_{o}})}^{2}\sum _{{n}_{1},{n}_{2}}\frac{{\cos }^{2}(\frac{{n}_{1}\,\pi }{a}x^{\prime} ){\cos }^{2}(\frac{{n}_{2}\,\pi }{a}y^{\prime} )}{{[(1-\delta ){n}_{1}^{2}+(1+\delta ){n}_{2}^{2}]}^{2}-{[(\omega -i\gamma )/{\omega }_{o}]}^{2}}.$$

Here ${\omega }_{o}=\sqrt{B/\rho h}\,\cdot \,(\pi /a)$ given by the dispersion relation of Eq. (2) has been used for clearer presentation later. For brevity, the parameters *x*ʹ and *y*ʹ denoting the driving point position of plate are omitted in the arguments of vibrating wave function ${\rm{\Psi }}(x,y\,\,;\omega )$ in Eq. (9). A more explicit derivation for the expression of ${\rm{\Psi }}(x,y\,\,;\omega )$ is provided in the section Methods.

**Figure 2:**
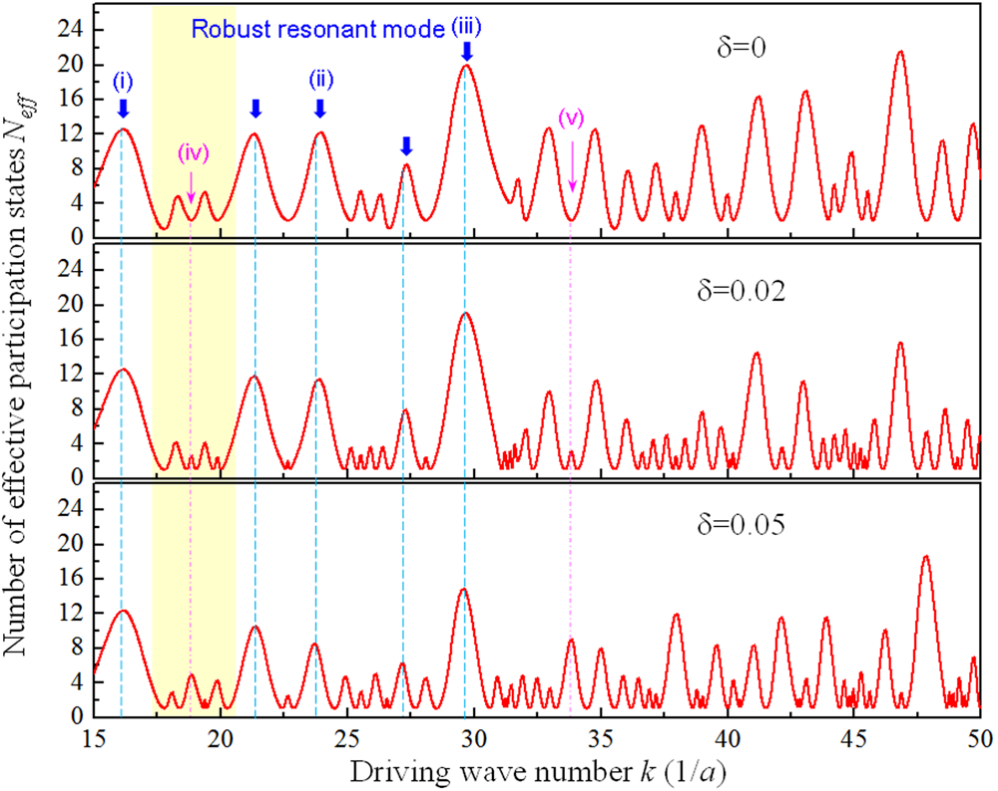
Effective participation mode number spectra *N*_*eff*_. The *N*_*eff*_ spectra for square plates with different symmetry-breaking parameters of *δ* = 0, 0.02, and 0.05 were calculated and shown from top to bottom. The redistribution of resonant peaks with increasing symmetry breaking manifests two types of resonant modes for the plate: one is the so-called robust mode (see the cases i-iii) with its peak position remaining nearly unchanged under orientation symmetry breaking; the other is the new-formed resonant mode occurring at the local minimum of the isotropic spectrum (see the cases of iv and v).

With the analytical approximation for the response modes, influences of orientation symmetry breaking on resonant spectra and wave functions of modern Chladni system can be explicitly analyzed. For a vibrating plate coupled to a driving point source, the resonance conditions can be quantitatively determined by analyzing the spectrum of effective radiated power efficiency at the driving point $(x^{\prime} ,y^{\prime} \,)$. The driving-point radiated power of vibrating plate has been confirmed to be directly proportional to the number of effective participated eigenstates *N*_*eff*_ in the response wave function^24^. It is worthy to note that *N*_*eff*_ for the vibrating plate is similar to the concept of acoustic density of states whose increment has been proved to play an important role for the enhancement of acoustic emission^25^. Since entropy is a logarithmic measure of the number of eigenmodes with significant participated probability in the coherent superposition to form the response wave function, the *N*_*eff*_ spectrum of the vibrating plate can be related to the entropy *S* as ${N}_{eff}(k)=\exp (S(k))\,$. A more detailed discussion to calculate the entropy corresponding to a given driving wave number by the weighting coefficient function in Eq. (10) is provided in the section Methods. In order to compare with the results of isotropic plate in previous works more directly, the argument of the expansion coefficient function in Eq. (10) has been changed from frequency *ω* to wave number *k* by simply using the dispersion relation with $\omega =C\cdot {k}^{2}$, where the coefficient *C* can be evaluated by Eqs (2 and 3) once the orientation angle *θ* is determined. By specifying the local maxima of *N*_*eff*_ spectrum under different *δ* parameters, the redistribution of resonant peaks of vibrating plates with orientation symmetry breaking can be analyzed. Figure 2 shows the calculated results of ${N}_{eff}(k)\,$ for the square plates with symmetry breaking parameters *δ* to be 0, 0.02, and 0.05. The calculated ${N}_{eff}(k)\,$ spectra can be seen to behave as oscillatory functions whose peak positions correspond to resonant wave numbers that leads the acoustic power transferred efficiency of system to be local maxima. The validity of determining the resonant peak positions by the maximum *N*_*eff*_ (or the maximum entropy) may be understood via the concept of energy equipartition in statistical mechanics, i.e. the more the eigenstates participating in the total energy configuration, the higher the energy it can possess since each eigenstate can offer the same energy contribution to the system. This so-called maximum entropy principle has been widely confirmed to be feasible and reliable to predict the collective behavior in multimode systems such as maximum emission for lasers^26^, self-organization for complex systems^27^, wave function localization for disordered systems^28^, and phase transitions for open quantum systems^29^. From the results of the redistributed *N*_*eff*_ spectra, some resonant modes (marked by blue downward arrows in Fig. 2) can be found to be so robust with their resonant peak positions remain almost unchanged when the symmetry-breaking parameter *δ* increases. These nearly unaffected peak positions can be seen to correspond to relatively larger *N*_*eff*_ (larger density of states) whose positions are mainly determined by the energy level distribution of the plate. Since the perturbation effect like the orientation symmetry breaking is insufficient strong to considerably shift the positions of clustered energy levels of the system, the number of participated eigenstates in the robust modes only decreases a little as *δ* increases. In addition, because the dominant participated eigenmodes in the coherent superposition can still have relatively high participated probability under the orientation symmetry breaking, the morphologies of the robust modes can be conjectured to be almost the same. A detailed analysis for the dependence of morphology variation on the eigenstate composition for the resonant modes will be discussed later. On the other hand, some new resonant peaks can be found to emerge at the positions of local minima for the isotropic case as the symmetry-breaking parameter increases. Once *δ* is sufficient large, *N*_*eff*_ for the new resonant modes can even exceed those of the original resonant modes in the isotropic case to become locally dominant states as seen the marks (iv) and (v) in Fig. 2.

**Figure 3:**
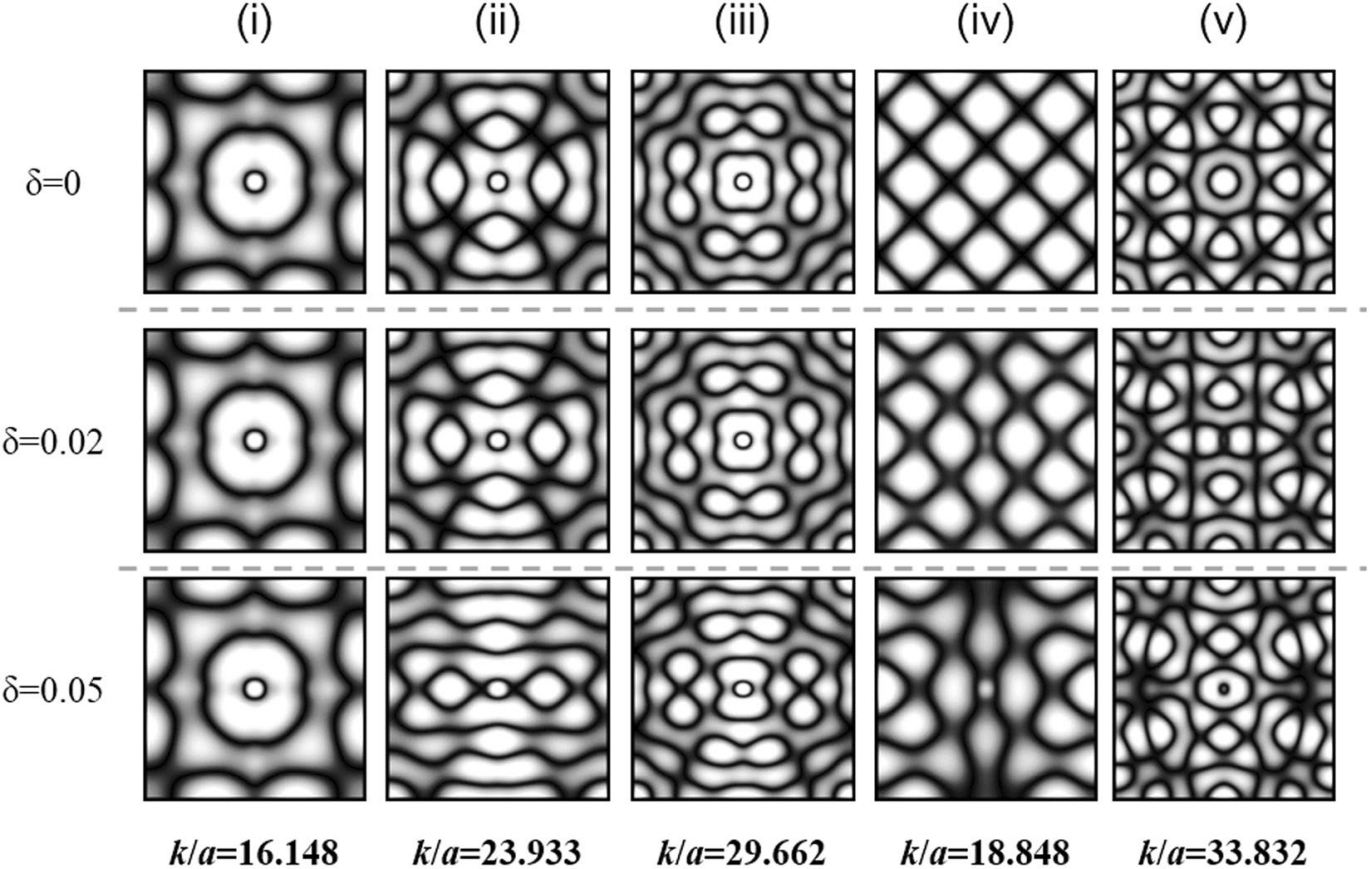
Calculated resonant wave functions of square plates. Resonant wave functions of square plates corresponding to the peaks (i)–(v) in Fig. 2 were calculated under different symmetry-breaking parameters *δ* to be 0 (top), 0.02 (middle), and 0.05 (bottom). The robust modes (i)–(iii) can be seen to only show slightly deformed morphologies under the orientation symmetry-breaking effect. In contrast, the morphologies of new resonant modes (iv) and (v) under non-zero symmetry breaking can be found to be dramatically different from the wave patterns of the isotropic cases

To examine the influences of orientation symmetry breaking on the wave patterns, the resonant wave functions of vibrating plates corresponding to driving wave numbers marked by (i)–(v) in Fig. 2 under different symmetry-breaking parameters *δ* = 0, 0.02, and 0.05 are calculated by Eqs (9–11) and shown in Fig. 3. Consistent with the aforementioned discussion, the overall structures of mode patterns for the robust modes (i)–(iii) remain nearly unchanged but only deform slightly along one coordinate axis as the symmetry-breaking parameter increases. The numerical results validate the fact that the larger the *N*_*eff*_ for the coherent superposition, the more stable the structure of the resonant mode against the perturbation. In contrast, the new resonant modes (iv) and (v) with non-zero *δ* can be obviously found to show totally different morphologies in comparison with the isotropic cases. Unlike typical plate wave functions with a presumable antinode at the driving point because that this position serves as the main excitation source for the plate vibration, it can be intriguingly seen that the driving position will turn into a nodal point in some new resonant modes under orientation symmetry breaking^16^.

**Figure 4:**
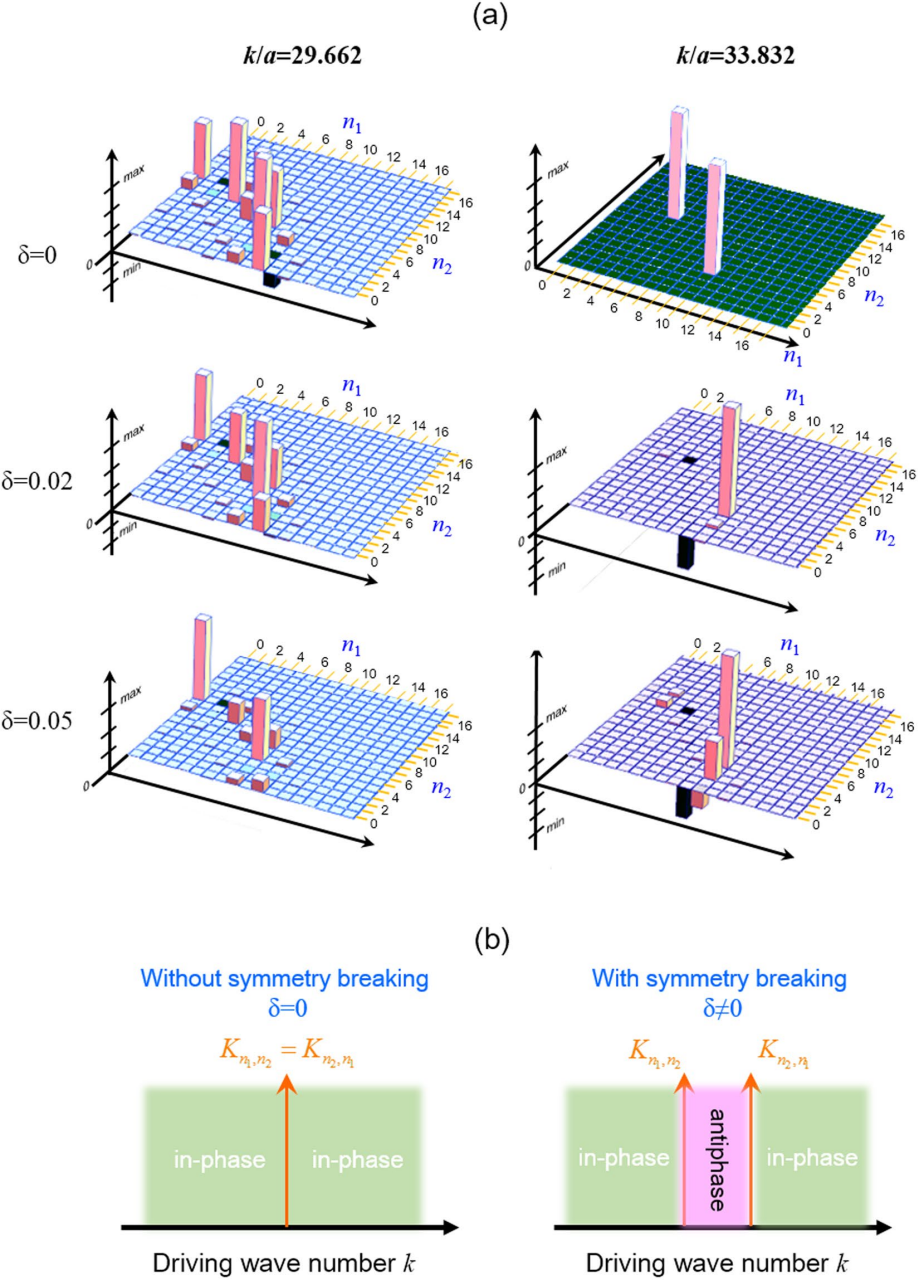
Studying mode pattern variation by mode composition analysis. (**a**) The eigenmode compositions for the cases of the robust mode (*k*/*a* = 29.662) and the new resonant mode (*k*/*a* = 33.832) under different symmetry-breaking parameters of *δ* = 0, 0.02, and 0.05 were analyzed and plotted from top to bottom. The main contributed eigenmodes for the robust mode can be found to be in-phase no matter how *δ* changes, while the dominant eigenmodes for the new resonant mode are clearly seen to be antiphase once *δ* becomes non-zero. (**b**) Conceptual diagram for explaining the origin of antiphase superposition from symmetry breaking. Once two degenerate eigenstates have been split due to orientation symmetry breaking, the anti-phase superposition will naturally occur when the driving wave number is tuned to in between the split levels ${K}_{{n}_{1},{n}_{2}}$ and ${K}_{{n}_{2},{n}_{1}}$.

In order to analyze the morphology transition with increasing elastic anisotropy more quantitatively, the eigenmode compositions given by the weighting coefficient ${C}_{{n}_{1},{n}_{2}}(k)\,$ for the cases of the robust resonant mode (*k*/*a* = 29.662) and the new resonant mode (*k*/*a* = 33.832) are further analyzed (Fig. 4a). For the case of robust mode, the number of eigenstates with significant participated probability can be seen to decrease a little as *δ* increases, which agrees with the results in Fig. 2. However, the slight decrement of *N*_*eff*_ for the robust mode does not influence the global morphology of the wave pattern because the significant participated eigenmodes still have sufficient large contribution to the coherent superposition. The slightly deformed wave patterns along one of the coordinate direction for the robust mode can be explained by the enlarged magnitude differences on the weighting coefficients for participated eigenmodes ${\psi }_{{n}_{1},{n}_{2}}$ and ${\psi }_{{n}_{2},{n}_{1}}$ as the symmetry-breaking parameter *δ* increases. Nevertheless, it can be clearly found that most of the dominant eigenstates in the robust modes remain in-phase in the superposition no matter how the symmetry-breaking parameter increases. On the contrary, the participating eigenmodes ${\psi }_{{n}_{1},{n}_{2}}$ and ${\psi }_{{n}_{2},{n}_{1}}$ for the new resonant mode show abrupt change from in-phase to antiphase superposition once there exists non-zero symmetry breaking. The antiphase superposition of eigenmodes has been known to be the main cause for wave patterns with a nodal point at the fixed or driving position for the free-edge plates^22^. However, the relationship between symmetry breaking and the presence of antiphase superposition in plate systems is seldom discussed with an explicit model so far. Using the developed analytical expression for the resonant mode, antiphase superposition induced by orientation symmetry breaking can be easily explained with the conceptual diagram (Fig. 4b). Without symmetry breaking (${K}_{{n}_{1},{n}_{2}}={K}_{{n}_{2},{n}_{1}}$), all degenerate eigenmodes ${\psi }_{{n}_{1},{n}_{2}}$ and ${\psi }_{{n}_{2},{n}_{1}}$ are in-phase to correspond to either positive or negative weights in the superposition no matter the driving wavenumber is larger or smaller than the closest eigenvalue ${K}_{{n}_{1},{n}_{2}}$. In contrast, once the symmetry breaking causes the degenerate level splitting, antiphase superposition naturally appears as long as the driving wave number is in between the split eigenvalues ${K}_{{n}_{1},{n}_{2}}$ and ${K}_{{n}_{2},{n}_{1}}$. From the other viewpoint, it is the degenerate level splitting to lead to the emergence of new local maximum in ${N}_{eff}\,$ spectrum so as to form the new resonant mode. Next the modern Chladni experiment of vibrating orthotropic plates is performed to confirm the developed theory.

### Experimental verification by orthogonal brass plates

**Figure 5:**
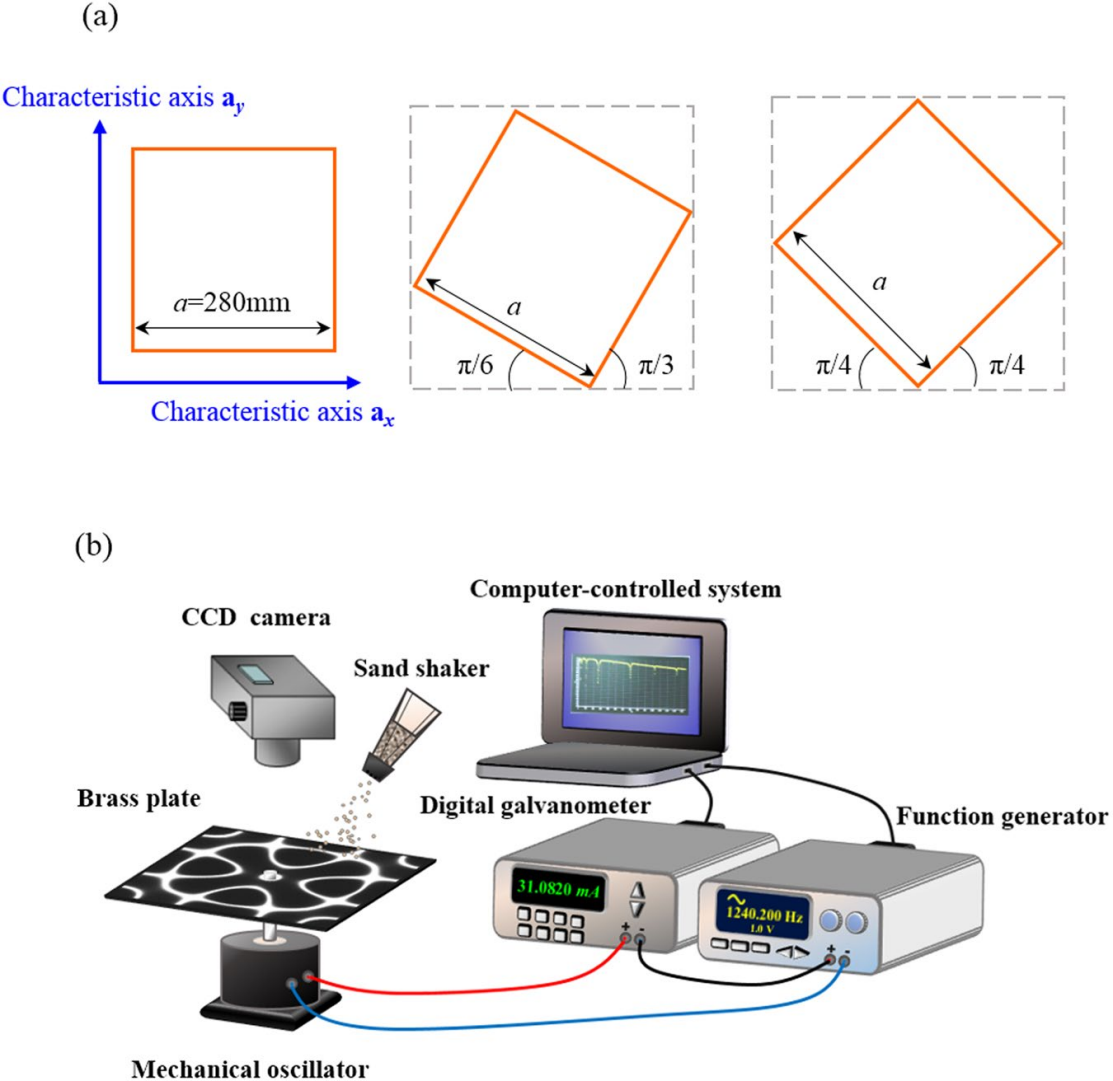
Setup of modern Chladni experiments with symmetry breaking. (**a)** Fabrication of thin plates with different magnitude of elastic anisotropy. Based on Eq. (3), the brass sheet was cut into three squares with their sides along the directions with angles of 0, π/6, and π/4 with respect to one of the characteristic axes of the orthotropic brass. (**b**) Apparatus for measuring the resonant spectrum and the corresponding nodal-line patterns for modern Chladni systems. Following the measurement in ref.^24^, a digital galvanometer was connected in series to the vibrating system (thin plate and mechanical oscillator) to probe the driving power efficiency for characterizing the resonant spectrum. The resonant Chladni figures were then recorded by the traditional sprinkling-sand approach.

**Figure 6:**
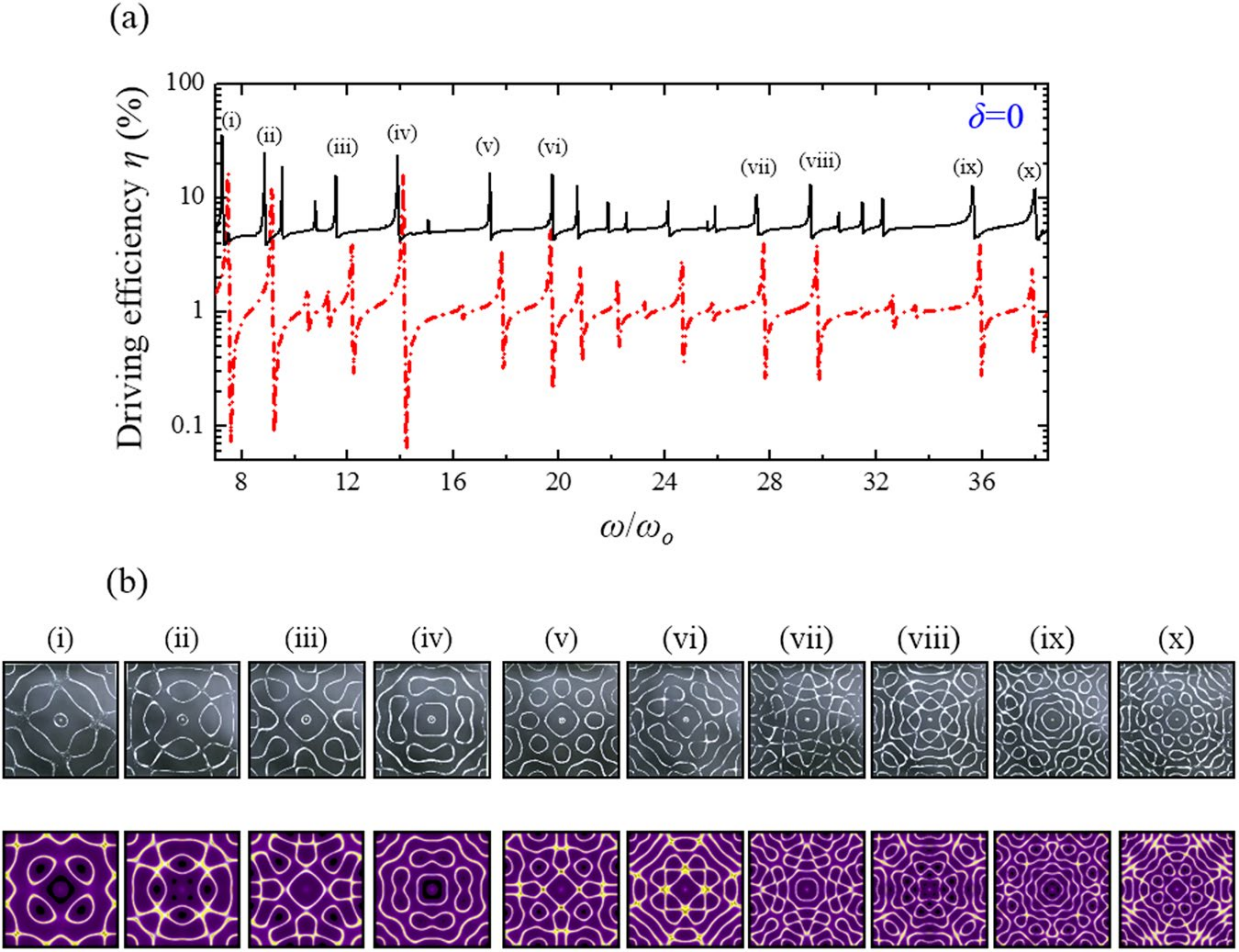
Experimental V.S. theoretical results for the square plate with *δ* = 0. (**a**) The experimental result (solid black line) and the theoretical reconstruction (red chain line) of the frequency spectra of the driving efficiency of power delivery *η* for the square brass plate with the cutting angle *θ* = π/4 and the symmetry-breaking parameter *δ* = 0. (**b**) The experimental results (first row) and the theoretical reconstructions (second row) of resonant Chladni figures corresponding to peaks (i)–(x) in the resonant spectrum. The high similarity between these results and those in ref.^24^ for the aluminum plate implies that the square brass plate with cutting angle *θ* = π/4 can be regarded as an isotropic system as the theoretical prediction.

**Figure 7:**
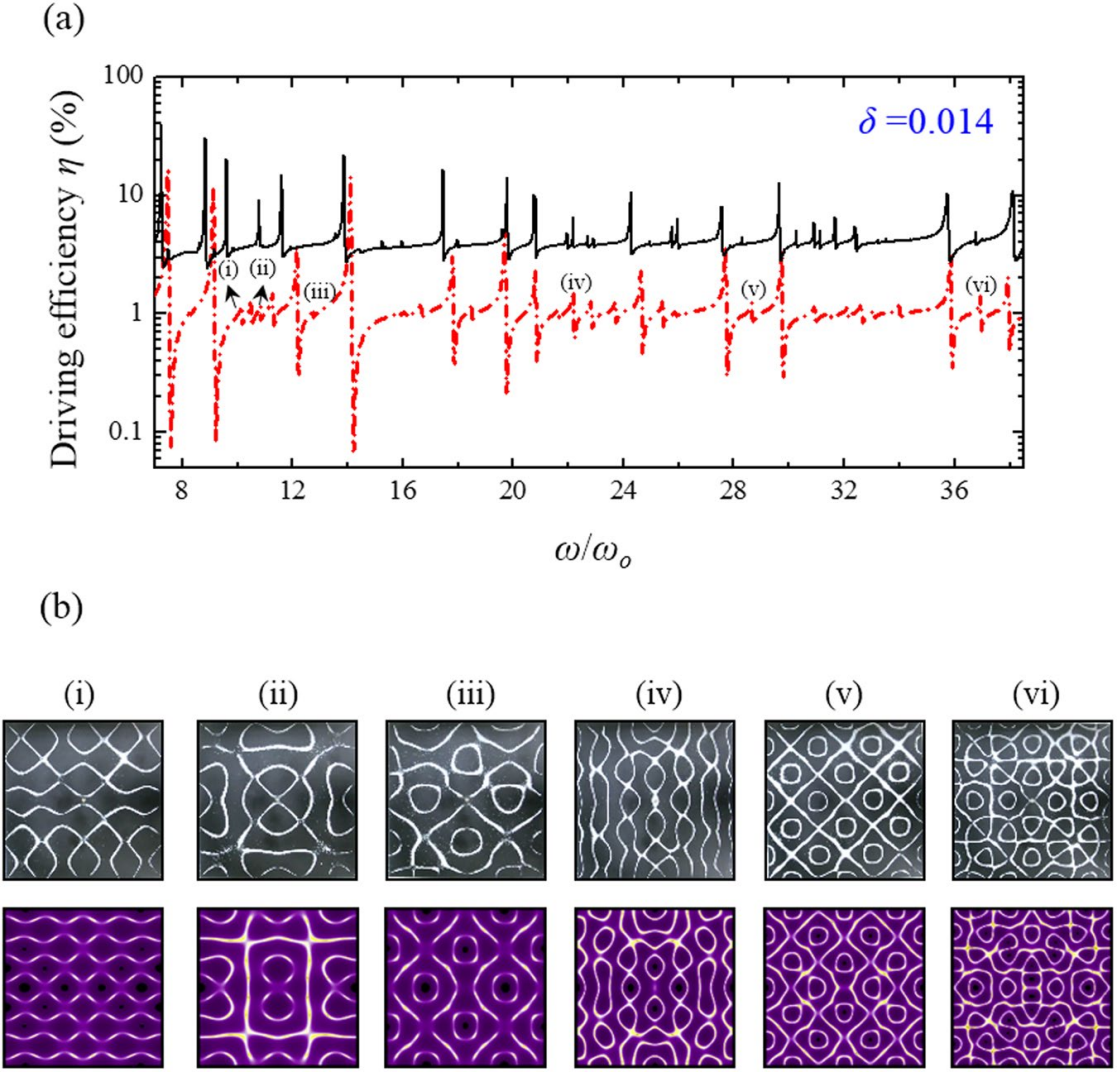
Experimental V.S. theoretical results for the square plate with *δ* = 0.014. (**a**) The experimental result (solid black line) and the theoretical reconstruction (red chain line) of the frequency spectra of the driving efficiency of power delivery *η* for the square brass plate with the cutting angle *θ* = π/6 and the symmetry-breaking parameter *δ* = 0.014. (**b**) The experimental results (first row) and the theoretical reconstructions (second row) of the resonant Chladni figures corresponding to peaks (i)–(vi) in the resonant spectrum. Compared with the results shown in Fig. 6(a,b), some new resonant peaks can be clearly seen to emerge from the orientation symmetry breaking. The Chladni figures of new resonant modes reveal morphologies with a nodal point at the driving position which is always an antinode in the isotropic plates.

**Figure 8:**
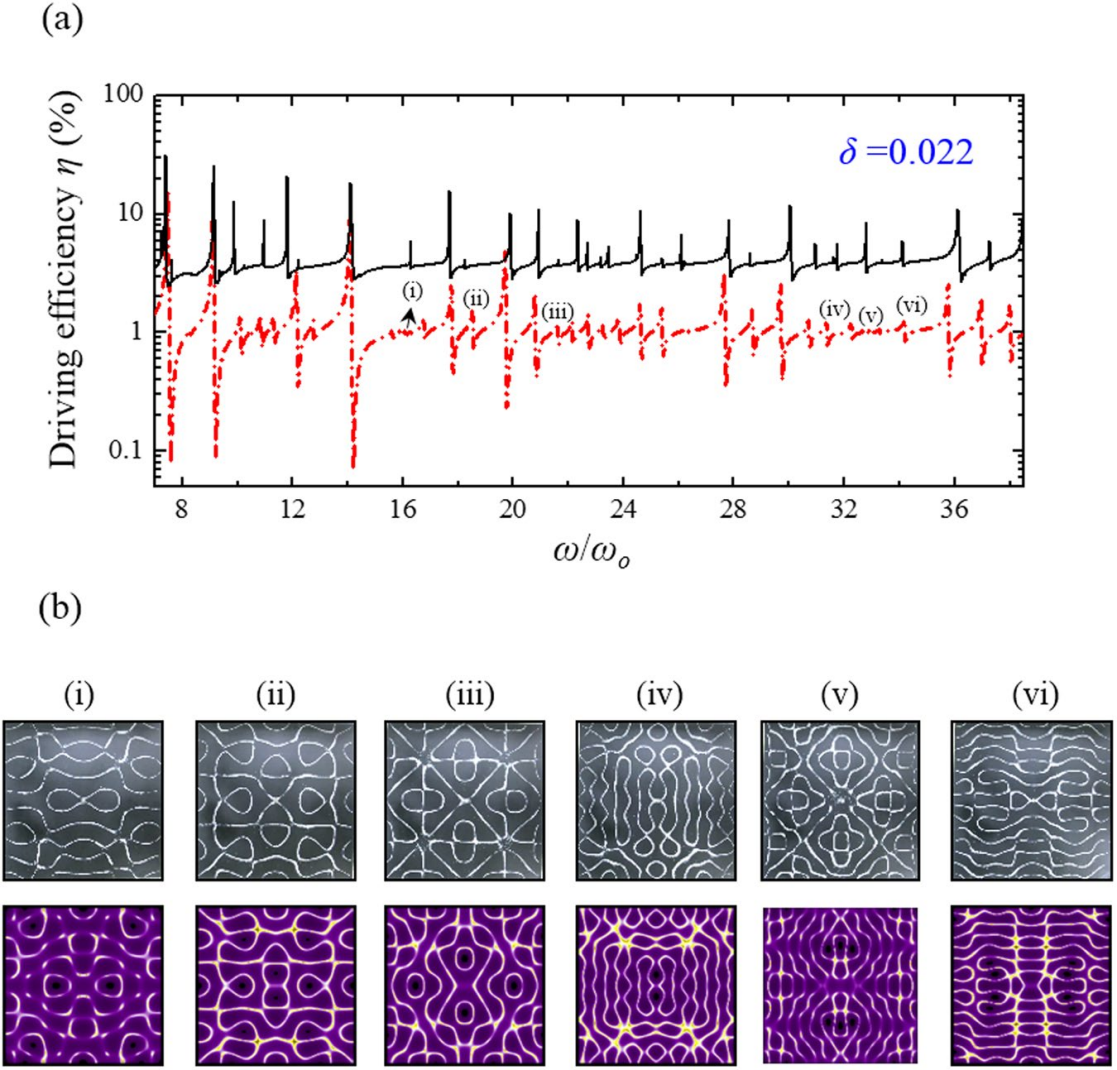
Experimental V.S. theoretical results for the square plate with *δ* = 0.022. (**a**) The experimental result (solid black line) and the theoretical reconstruction (red chain line) of the frequency spectra of the driving efficiency of power delivery *η* for the square brass plate with the cutting angle *θ* = 0 and the symmetry-breaking parameter *δ* = 0.022. (**b**) The experimental results (first row) and the theoretical reconstructions (second row) of the resonant Chladni figures corresponding to peaks (i)–(vi) in the resonant spectrum. Compared with the results shown in Fig. 7(a,b), some new resonant modes can be seen to become locally dominant states as the symmetry breaking parameter increases further. These new dominant modes can be found to reveal deformed morphologies along one of the coordinate axes and with a nodal-point driving position (see the cases of i, iv, and iv).

Because of its appropriate stiffness and elastic properties^21^, the orthotropic brass which plays important roles in industry and musical instrument manufacturing was utilized for the modern Chladni experiment. To create thin plates with different elastic anisotropy corresponding to different symmetry-breaking parameters *δ*, a brass sheet with a thickness of 0.8 mm were cut into three squares with the side-length *a* = 280 mm and with their sides along the cutting direction in rotation angles *θ* of 0, π/6, and π/4 with respect to one of the characteristic axes of brass (Fig. 5a). All brass plates were fixed and driven at the square center as seen the experimental setup for modern Chladni figures (Fig. 5b). The solid black lines in Figs 6a–8a show the experimental frequency spectra of the driving efficiency of power delivery *η* for the brass plates with *θ* = π/4, π/6, and 0, respectively. According to the orientation-dependent dispersion relation given by Eq. (3), it can be easily deduced that the symmetry-breaking parameter increases as the cutting angle *θ* deviates away from *θ* = π/4, i.e. $\delta (0)\, > \delta (\pi /6) > \delta (\pi /4)$. Consistent with previous theoretical discussion, several new resonant peaks can be clearly found in the frequency spectra as the symmetry breaking parameter increases (Figs 6a–8a). Subsequently, Chladni nodal-line patterns corresponding to the resonant modes of vibrating brass plates were recorded by using the traditional method. The first row of Fig. 6b shows the experimental Chladni figures corresponding to the resonant peaks (i)–(x) in Fig. 6a of the brass plate with *θ* = π/4. These resonant modes exactly belong to the robust modes whose resonant peak positions can be clearly seen to be almost unchanged with the symmetry breaking (Figs 6a–8a). Besides, resonant Chladni figures of these robust modes for the case of *θ* = π/4 can be seen to present highly symmetric morphologies that are quite similar to the results for the isotropic square plate^24^. The comparatively less resonant peaks in the frequency spectrum and the high-symmetry nodal-line patterns of resonant modes implies the brass square plate with *θ* = π/4 can be certainly viewed as an isotropic system with *δ* = 0. The first rows of Figs 7b and 8b show Chladni figures corresponding to some new resonant modes marked by (i)–(vi) in Figs 7a and 8a. All these new resonant modes induced by orientation symmetry breaking indeed reveal nodal patterns with the driving position to be a nodal point as the theoretical prediction. Moreover, some Chladni figures of the new resonant modes can also be found to present deformed morphologies that break the reflection symmetry with respect to the square diagonals when the symmetry breaking parameter increases even further (see i, iv, and vi in Fig. 8b).

### Reconstructing experimental resonant modes by developed model

For validating its feasibility to analyze modern Chladni systems with orientation symmetry breaking, the developed model is further exploited to reconstruct all the experimental observations subsequently. Theoretically, the driving efficiency of power delivery *η* of the vibrating plate can be expressed as the square of the ratio of the reaction amplitude $\alpha \,{\rm{\Psi }}(x^{\prime} ,y^{\prime} ;\omega )$ to the driving amplitude *Q* that can be explicitly derived as^16^

12 $$\eta (\omega )={[\frac{|\alpha {\rm{\Psi }}(x^{\prime} ,y^{\prime} ;\omega )|}{Q}]}^{2}={(\frac{{m}_{d}}{{m}_{p}})}^{2}|\frac{\alpha \,{\rm{\Xi }}(x^{\prime} ,y^{\prime} ;\omega )}{1+\alpha \,{\rm{\Xi }}(x^{\prime} ,y^{\prime} ;\omega )}|.$$

**Figure 9:**
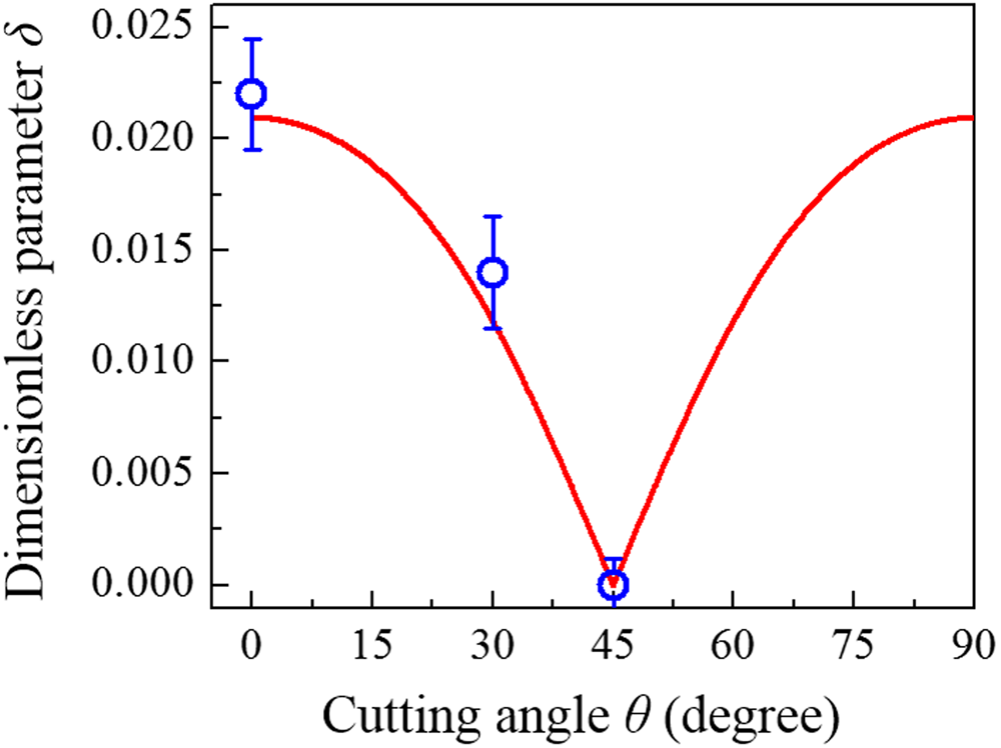
Orientation-dependent symmetry-breaking parameter *δ* for the brass plate. The dependence of symmetry-breaking parameter on the orientation of brass plate was evaluated by Eq. (3) with the elastic constants^21^ (red line) to compare with the results from reconstructions (blue hollow circles). The error bars were determined by the allowable range of *δ* in fitting the experimental resonant spectra and Chladni figures for a batch of brass square plates fabricated with the same cutting angles.

Utilizing Eq. (12) with the parameters of *α* = 0.1, *ω*_*o*_ = 104 *s*^−1^, *γ* = 0.16*ω*_*o*_, and *m*_*d*_/*m*_*p*_ = 0.1, the reconstructed frequency spectra of driving efficiency are calculated and shown by the red chain lines in Figs 6a–8a. Note that the damping coefficient *γ* and coupling factor *α* that are respectively associated with the widths and positions of resonant peaks can be directly determined by best fitting the numerical calculation to the experimental results. By fine tuning the symmetry-breaking parameter *δ* in the calculation, the overall structures of experimental frequency spectra can be seen to be nicely matched by the numerical results. The best fitting between the experimental and numerical spectra in Figs 6a–8a correspond to symmetry-breaking parameters *δ* to be 0, 0.014, and 0.022 for the brass plates with cutting angles *θ* of π/4, π/6, and 0, respectively. Using Eqs (9 and 10) with the driving frequencies to be at the peak positions marked in Figs 6a–8a, the corresponding Chladni figures for the brass plates can be reconstructed by evaluating the inverse of wave patterns ${|{\rm{\Psi }}(x,y;\omega )|}^{2}$ as seen in the second rows of Figs 6b–8b. Even though slight differences can be found in detailed structures due to the linear approximation in theory and the manufacturing imperfections of plates, the global morphologies of experimental Chladni figures are satisfactorily reconstructed by the numerical patterns of current model. The good agreement between the numerical reconstructions and the experimental results once again verifies the applicability of the developed model to nicely approximate the resonant behavior of vibrating thin plates subject to orientation symmetry breaking. Finally, the orientation-dependent symmetry-breaking parameter for the brass plate given by Eq. (3) is further calculated with the elastic constants^21^ of *E*_*x*_ = 107.7 GPa, *E*_*y*_ = 126.5 GPa, and ${\nu }_{xy}\,{E}_{y}+2G$ = 80.3 GPa to compare with the results from reconstructions (Fig. 9). The high consistency between the reconstructing parameters and the results calculated from the elastic theory further verifies that the developed model can be a powerful tool to be combined with the numerically modal-expansion method^30^ to analyze the anisotropic elastic constants of orthotropic plates more efficiently.

## Discussion

In this study, point-driven modern Chladni systems subject to the orientation symmetry breaking effect have been theoretically and experimentally explored in depth. By cutting the orthotropic brass sheet into squares with their sides in rotation angles with respect to the characteristic axes, vibrating plates with different elastic anisotropy have been systematically explored. It has been confirmed that the resonant spectra reveal explicit redistribution and occurrence of new resonant modes under the orientation symmetry breaking effect which leads the degenerate level splitting of the orthotropic plates. More intriguingly, the driving position in some new resonant modes has been found to turn into a nodal point, whereas this position is always an antinode in the isotropic plates. Using the analytical model developed by including a dimensionless parameter to consider the orientation symmetry breaking of plate in a generalized manner, formation of the peculiar morphologies of new resonant modes from the antiphase superposition has been unambiguously resolved. Furthermore, the developed model has been utilized to reconstruct all experimental observations of resonant spectra and resonant Chladni figures subject to orientation symmetry breaking with high consistency. The good agreement between the theoretical reconstructions and experimental results not only proves the feasibility of the developed model to describe point-driven Chladni systems with orientation symmetry breaking but also provide a powerful tool to use the analytical model to analyze important elastic constants of orthotropic plates in a more time-saving way.

## Methods

### Response wave function of modern Chladni plates

According to ref.^16^, the response wave functions $\Psi (x,y;\omega )$ of a vibrating thin plate coupled with a sinusoidal point source with oscillation frequency *ω* at $(x^{\prime} ,y^{\prime} \,)$ can be governed by the inhomogeneous bi-harmonic equation:

13 $$({\nabla }^{4}-\frac{\rho \,h\,{\omega }^{2}}{D}){\rm{\Psi }}(x,y\,;\omega )=\frac{{m}_{d}\,{\omega }^{2}}{D}[Q-\alpha (\frac{{m}_{p}}{{m}_{d}}){\rm{\Psi }}(x^{\prime} ,y^{\prime} ;\omega )]\delta (x-x^{\prime} )\delta (y-y^{\prime} ),$$

where ${\nabla }^{4}$ is the bi-harmonic operator; *D* is the flexural rigidity; *ρ* is the mass density of plate; *h* is the plate thickness; *m*_*d*_ and *m*_*p*_ are respectively the mass of driving oscillator and thin plate; *Q* is the amplitude of driving oscillator; $\alpha \in [0,1]$ is the dimensionless coupling factor which describes the coupling strength between the plate and the driving oscillator. By using the complete set of eigenfunctions ${\psi }_{n}(x,y)$ and eigenvalues *ω*_*n*_ given by the homogeneous equation $({\nabla }^{4}-\rho h{\omega }_{n}^{2}/D){\psi }_{n}(x,y)=0$, Eq. (13) can be solved to lead to

14 $${\rm{\Psi }}(x,y;\omega )=[Q(\frac{{m}_{d}}{{m}_{p}})-\alpha \,{\rm{\Psi }}(x^{\prime} ,y^{\prime} \,;\omega )]\,\sum _{n}\frac{A\,{\omega }^{2}}{({{\omega }_{n}}^{2}-{\omega }^{2})}\,{\psi }_{n}^{\ast }(x^{\prime} ,y^{\prime} \,){\psi }_{n}(x,y),$$

where *A* is the area of plate and *m*_*p*_ = *ρhA*. Note that $\delta (x-x^{\prime} )\delta (y-y^{\prime} )={\sum }_{n}{\psi }_{n}^{\ast }(x^{\prime} ,y^{\prime} \,){\psi }_{n}(x,y)$ and $\Psi (x,y;\omega )={\sum }_{n}{C}_{n}(\omega )\,\,{\psi }_{n}(x,y)$ have been used here. Equation (14) clearly reveals that the response wave function ${\rm{\Psi }}(x,y;\omega )$ is directly influenced by its response at the driving point ${\rm{\Psi }}(x^{\prime} ,y^{\prime} ;\omega )$ and thus manifests the so-called coupling effect. Setting $x=x^{\prime}$ and $y=y^{\prime}$ on both sides of Eq. (14), the self-consistent solution of the driving point response $\Psi (x^{\prime} ,y^{\prime} ;\omega )$ can be analytically derived as

15 $${\rm{\Psi }}(x^{\prime} ,y^{\prime} ;\omega )=Q(\frac{{m}_{d}}{{m}_{p}})\frac{{\rm{\Xi }}(x^{\prime} ,y^{\prime} ;\omega )}{1+\alpha \,{\rm{\Xi }}(x^{\prime} ,y^{\prime} ;\omega )},$$

where ${\rm{\Xi }}(x^{\prime} ,y^{\prime} ;\omega )$ is a dimensionless meromorphic function given by

16 $${\rm{\Xi }}(x^{\prime} ,y^{\prime} ;\omega )=\sum _{n}\frac{A\,{\omega }^{2}{|{\psi }_{n}(x^{\prime} ,y^{\prime} )|}^{2}}{{{\omega }_{n}}^{2}-{(\omega -i\gamma )}^{2}},$$

where the damping rate *γ* is considered by replacing *ω* with *ω* − *iγ*. The damping factor *γ* is closely related to the quality *Q*-factor of the resonance of the realistic thin plate system. In term of ${\rm{\Xi }}(x^{\prime} ,y^{\prime} ;\omega )$, the final form of the response wave function can be given by

17 $$\begin{array}{c}{\rm{\Psi }}(x,y;\omega )=\sum _{n}{C}_{n}(\omega )\,\,{\psi }_{n}(x,y)\\ \,\,\,\,\,\,=\sum _{n}[\frac{Q({m}_{d}/{m}_{p})}{1+\alpha {\rm{\Xi }}(x^{\prime} ,y^{\prime} ;\omega )}\cdot \frac{A\,{\omega }^{2}\,{\psi }_{n}^{\ast }(x^{\prime} ,y^{\prime} )}{{{\omega }_{n}}^{2}-{(\omega -i\gamma )}^{2}}]\,{\psi }_{n}(x,y).\end{array}$$

The analytical expression of the expansion coefficient ${C}_{n}(\omega )$ of Eq. (17) explicitly indicates the eigenmode composition in the coherent superposition under a given driving frequency *ω*.

### Effective number of participated eigenmodes in the coherent superposition

For the response mode contributed by the coherent superposition of numerous eigenmodes ${\psi }_{n}(x,y)$ as ${\rm{\Psi }}(x,y;k)={\sum }_{n}{C}_{n}(k)\,\,{\psi }_{n}(x,y)$, the number of effectively participating eigenmodes as a function of driving wave number *k* can be given by the entropy $S(k)$ of the system as ${N}_{eff}(k)=\exp (S(k))\,$. According to Shannon’s information theory^31^, the entropy of a coherent state can be written as $S(k)=-\,\sum _{n}{p}_{n}(k)\mathrm{ln}[{p}_{n}(k)]$, where ${p}_{n}(k)$ is the probability of a specific eigenmode *ψ*_*n*_ contributing to the driven response under a given wave number. According to the interpretation for quantum wave function, the probability can be expressed by the normalized expansion coefficient

18 $${c}_{n}(k)=\frac{{C}_{n}(k)}{\sqrt{\sum _{n}{|{C}_{n}(k)|}^{2}}}$$

as ${p}_{n}(k)={|{c}_{n}(k)|}^{2}$. Considering a coherent state that is composed by *N* eigenmodes with equal probabilities, i.e. ${p}_{n}=1/N$, the information entropy can be evaluated to be $S=\,\mathrm{ln}\,N$ whose exponential form can certainly give the number of effective participated eigenmodes.

### Measurement of resonant spectra and resonant Chladni figures of thin plates

The setup and processes for measuring modern Chladni figures at resonance are the same as those mentioned in refs^16,24^. To prepare thin plate systems with different elastic anisotropy corresponding to different symmetry-breaking parameters, the brass sheet with thickness of 0.8 mm was cut into squares with the side-length of 280 mm and with different cutting angles *θ* to be 0, π/6, and π/4 with respect to the characteristic axes of the orthotropic brass (Fig. 5a). The center of thin plate was fixed with a screw supporter that was driven by an electronically controlled mechanical oscillator with sinusoidal wave of variable frequency. The electronically controlling system consists of a function generator with its signal to be amplified to excite the mechanical oscillation and a digital galvanometer connected in series to the oscillator to probe the effective driving power of the whole plate system (Fig. 5b). From the frequency response of the measured driving power for total vibrating system (thin plate and mechanical oscillator) ${P}_{r}(\omega )$ as well as for the mechanical oscillator only ${P}_{o}(\omega )$, the driving efficiency of power delivery $\eta =[{P}_{o}(\omega )-{P}_{r}(\omega )]/{P}_{o}(\omega )$ can be analyzed to characterize the resonant spectrum of modern Chladni systems^16^. Subsequently, resonant Chladni figures can be recorded at the resonant frequencies resolved from the driving efficiency spectrum by using the traditional sprinkling-sand method.

### Data availability statement

All data generated or analyzed during this study are included in this published article.
